# Elevation of Alanine Aminotransferase Activity Occurs after Activation of the Cell-Death Signaling Initiated by Pattern-Recognition Receptors ‎but before Activation of Cytolytic Effectors in NK or CD8+ T Cells in the Liver During Acute HCV Infection

**DOI:** 10.1371/journal.pone.0165533

**Published:** 2016-10-27

**Authors:** Youkyung H. Choi, Nancy Jin, Fiona Kelly, SenthilKumar K. Sakthivel, Tianwei Yu

**Affiliations:** 1 Division of Viral Hepatitis, Centers for Disease Control and Prevention, Atlanta, Georgia, United States of America; 2 Department of Biostatistics and Bioinformatics, Rollins School of Public Health, Emory University, Atlanta, Georgia, United States of America; University of Thessaly Faculty of Medicine, GREECE

## Abstract

Pattern-recognition receptors (PRRs) promote host defenses against HCV infection by binding to their corresponding adapter molecules leading to the initiation of innate immune responses including cell death. We investigated the expression of PRR genes, biomarkers of liver cell-death, and T cell and NK cell activation/inhibition-related genes in liver and serum obtained from three experimentally infected chimpanzees with acute HCV infection, and analyzed the correlation between gene expression levels and clinical profiles. Our results showed that expression of hepatic RIG-I, TLR3, TLR7, 2OAS1, and CXCL10 mRNAs was upregulated as early as 7 days post-inoculation and peaked 12 to 83 days post-inoculation. All of the three HCV infected chimpanzees exhibited significant elevations of serum alanine aminotransferase (ALT) activity between 70 and 95 days after inoculation. Elevated levels of serum cytokeratin 18 (CK-18) and caspases 3 and 7 activity coincided closely with the rise of ALT activity, and were preceded by significant increases in levels of caspase 3 and caspase 7 mRNAs in the liver. Particularly we found that significant positive auto-correlations were observed between RIG-I, TLR3, CXCL10, 2OAS1, and PD-L1 mRNA and ALT activity at 3 to 12 days before the peak of ALT activity. However, we observed substantial negative auto-correlations between T cell and NK cell activation/inhibition-related genes and ALT activity at 5 to 32 days after the peak of ALT activity. Our results indicated cell death signaling is preceded by early induction of RIG-I, TLR3, 2OAS1, and CXCL10 mRNAs which leads to elevation of ALT activity and this signaling pathway occurs before the activation of NK and T cells during acute HCV infection. Our study suggests that PRRs and type I IFN response may play a critical role in development of liver cell injury related to viral clearance during acute HCV infection.

## Introduction

HCV infection is the most common blood-borne infection in the US, with approximately 3 million persons living with the infection [1]. Percutaneous exposure to contaminated blood is the most frequent mode of HCV transmission with injecting drug use (IDU) being the predominant risk factor for infection. Surveillance data show that from 2006–2012, there was a nationwide increase in reported cases of acute HCV infection, with the largest increases occurring east of the Mississippi River, particularly in the Appalachian states [2]. Nationwide, nearly 30,000 acute hepatitis C infections are estimated to have occurred in 2013. Demographic and behavioral data show further that the majority of cases were among young persons (aged ≤30 years) from nonurban areas with IDU as the principal risk factor [3].

Acute hepatitis C is defined by the presence of any sign or symptom of acute viral hepatitis plus either jaundice or elevated serum alanine aminotransferase (ALT) activity, with serologic evidence of anti-HCV antibody or HCV RNA within 6 months of exposure. In 2012, the case definition for acute HCV infection was expanded to include cases with negative HCV antibody in blood followed by positive antibody within 6 months [3]. However, most people with acute HCV infection at time of diagnostic testing are already positive for anti-HCV antibody.

Biomarkers of host response to HCV during the early phase of infection may be used as an adjunct to diagnose acute hepatitis C more definitively and to provide a sharper case definition for acute hepatitis C [4]. Host responses include those generated by innate and adaptive immunologic mechanisms and those relating to cell death signaling. Studying the chimpanzee model of HCV infection allows host responses to be analyzed closely, particularly during the early stage of infection.

In experimentally infected chimpanzees, HCV RNA can be detectable in blood in 1 to 2 weeks after inoculation and is maintained for an additional 8 to 18 weeks [5–9]. Serum ALT activity peaks between 6 and 10 weeks, when levels of HCV RNA begin to decline. ALT activity is an indicator of liver injury induced hepatocytic death. Two main mechanisms of cell death have been described; disordered necrosis and programmed apoptosis. Studies have shown that these two processes are part of the same death spectrum [10, 11]. Apoptosis is induced intrinsically by mitochondrial death and extrinsically following triggering of the cell death pathway by receptors such as the tumor necrosis factor (TNF)α, Fas ligand (FASL), and TNF-related apoptosis-inducing ligand (TRAIL) [11]. Cysteine-aspartate proteases called caspases, are activated at the end of both pathways, and may be classified into two major groups: initiator caspases (such as caspases 2, 8, 9, and 10), which confer regulatory function; and effector caspases (such as caspases 3, 6, and 7), which execute apoptotic cell death [12].

HCV activates pattern-recognition receptors (PRRs) including Toll-like receptors (TLRs) and retinoic acid-inducible gene 1 (RIG-I) very early after infection [13]. PRRs initiate mechanisms to eliminate viral infection as a part of the host innate immune response [14]. Strong host immune responses are induced days after HCV infection in chimpanzees in which many of the immune response genes are found to be IFN-stimulated genes (ISG) [5, 7–9]. Activation of ISG in the early phase of acute HCV infection are initiated by detecting viral pathogen associated molecular patterns (PAMPs) through PRRs. PRR-induced responses are involved in the production of pro-inflammatory cytokines, type I and type III interferons (IFNs), which together act to limit viral replication by initiating cell death [15–21]. Exact mechanisms of interaction between IFN induction and apoptosis are unclear. However, it is known that TLR proteins recognize RNA as ligand and inducting type I and type III IFN responses [22, 23], for example, RIG-I, a cytosolic RNA helicase, that can recognize both double-stranded and single-stranded RNA can then induce a downstream signaling cascade that finally leads to apoptosis [24]. In addition, HCV-specific T cells and NK cells also have been shown to be associated with virus clearance and hepatic injury from apoptosis [25, 26].

We report here the kinetics of hepatic gene expression of markers of innate and adaptive immunity and of cell death signaling and the appearance of biomarkers in peripheral blood generated from such expression in HCV-infected chimpanzees during the acute phase. We show the markers of innate immunity and cell death signaling both in the liver and serum precede or coincide with rise of serum ALT activity, whereas the markers of adaptive immunity in the liver and the blood tend to occur after the peak of serum ALT activity.

## Materials and Methods

### Chimpanzees

This study was carried out in strict accordance with the recommendations in the Guide for the Care and Use of Laboratory Animals of the Centers for Disease Control and Prevention. The protocol was approved by the Institutional Animal Care and Use Committee of the Centers for Disease Control and Prevention (Protocol Number: 1363KRACHIC). The animals were anesthetized by the Animal Resource Branch personnel with tiletamine (telazole) for liver biopsy in the dose of 5mg/kg through intermuscular (IM), ketamine HCL was used to sedate for blood collection with 10 mg/kg dose through IM, and all efforts were made to minimize suffering. The diet consisted of a mixture of a high-protein chow (Lab Diet High-Protein Monkey Diet 5045, PMI Nutrition International, Saint Louis, MO), high-fiber chow (Fiber-Plus Monkey Diet 5049, PMI Nutrition International), various fruits and vegetables, and treats (Bio-serv, Frenchtown, NJ). Animals were singly and pair housed in accordance with federal laws, regulations, and the Guide for the Care and Use of Laboratory Animals. Prior to inoculation with hepatitis C and at the conclusion of the study, animals were pair housed. After inoculation with hepatitis C, animals were housed individually (the approximate floor area was over 25.1 sq ft) to prevent viral transmission but were allowed visual and auditory contact with one another. Environmental enrichment consisted of social (e.g. pair housing and human interaction), structural (e.g. swings, perches, tunnels), manipulanda (e.g. various toys rotated on a weekly or biweekly basis), sensory (e.g. visual, tactile, and olfactive such as television, music, videos, etc.), various novel food items, and various foraging opportunities. Analgesic used for pain relieve was injectable and oral meloxicam (Metacam, Boehringer Ingelheim, St Joseph, MO) administered intramuscularly immediately after the liver biopsy and orally on subsequent days of treatment at a dosage of 0.3mg/kg. The study was done between Aug, 2005 and March, 2006. Three chimpanzees (CH1541, CH256, and CH6413) were inoculated intravenously with HCV genotype 1a. The inocula varied from 10^3^ to 10^3.5^ chimpanzee infectious doses (CID) [27–29]. Serum samples were tested at baseline and weekly after inoculation for HCV RNA using the Cobas Amplicor system according to manufacturer’s recommendation (detection limit: 600 IU/ml) (Roche Molecular Systems, Pleasanton, CA) and further quantified using the Amplicor Monitor system (Roche Molecular Systems). Anti-HCV IgG was measured using the ORTHO 3.0 ELISA test system (Ortho-Clinical Diagnostics, Raritan, NJ). ALT activity was measured using the assay manufactured by Drew Scientific (Dallas, TX) and the cut-off value for each chimpanzee was set as 3 standard deviations above the mean of 9 or 10 pre-inoculation serum values.

### Gene expression of apoptosis, innate, and adaptive immune response markers in liver and PBMC specimens

Weekly liver needle biopsies were obtained at baseline and after HCV inoculation. Total RNA was extracted from liver biopsy samples and PBMCs using the TRIzol Plus RNA purification kit according to manufacturer’s recommendation (Ambion, Austin, TX). cDNA was prepared using 100 ng of total RNA by a high-capacity reverse transcription kit (Applied Biosystems, Foster City, CA) and used as template for real-time PCR performed using the SYBR green PCR JumpStart Master Mix (Sigma, St. Louis, MO) for caspase-3, caspase-7, caspase-8, caspase-9, caspase-10, and APAF-1 transcripts. Transcripts of 2OAS-1, RIG-1, TLR3, TLR7, TNFα, CXCL10, perforin, CXCR3, CCR1, CCR7, NKG2D, KIR2D, CD8β IFNγ, CD86, PD-1, PD-L1, CTLA-4, and Tim-3 in liver, and PBMCs were analyzed by Taqman real-time PCR using Applied Biosystems 7900HT Fast Real-time PCR system (Life Technologies, Grand Island, NY). Information about sequences of primers and probes is described in Supporting materials (S1 Materials and Methods). Greater than two-fold changes (2^-ΔΔ^CT) in altered gene expression were considered to be significant. Each gene’s mRNA level was normalized to an endogenous reference gene and expressed as an increase over baseline liver tissue levels.

### Cytokeratin-18 and caspase 3/7 activity in serum

The apoptosis-mediated neoepitope (M30 antigen) located in the C-terminal domain of cytokeratin-18 (CK-18) (amino acid 387–396) was measured by the M30-Apoptosense ELISA assay (Peviva AB, Bromma, Sweden). Caspase 3 and 7 activities in serum were measured by Caspase-Glo (Promega, Madison, WI). For both assays, cut-off values were calculated from 10 baseline specimens from each chimpanzee and set as the mean plus 1.6 times the standard deviation. All assays were performed in duplicate.

### Chemokines, and soluble PD-1 in serum/plasma specimens

Serum levels of CXCL10 were measured using Chemokine Array ELISA kit (RayBiotech, Norcross, GA). Soluble PD-1 (sPD-1) was measured in serum or plasma using an in-house enzyme-linked immunosorbent assay (ELISA), the details of which are included in the Supporting Information. Cut-off values for sPD-1 were calculated for each chimpanzee from 10 serum samples obtained before HCV inoculation (CH6413: 0.17 ng/ml, CH256: 0.22 ng/ml, and CH1541: 0.12 ng/ml). Background levels of sPD-1, determined from 24 plasma samples obtained from historical control specimens of 3 naïve chimpanzees, were found not to exceed 0.1 ng/ml.

### Statistical analysis

To investigate the relationship between hepatic mRNA expression, serum protein levels, serum HCV RNA titer, and ALT activity during acute HCV infection, Pearson and lag correlation tests were performed. Mean values of observed data from all three animals were used due to the consistency and similarity of HCV RNA concentration and ALT profiles in each animal. The Pearson correlation test was performed using SPSS software version 21 (IBM Inc. Armonk, NY) and the Comprehensive R Archive Network (CRAN), Bioconductor (R software version 3.2.2) [30] was used for the lag correlation tests. Lagged temporal correlations were calculated between each biomarker and ALT activity after shifting expression data to align with the peak of ALT activity [31]. Linear interpolation was applied when necessary because of the occurrence of missing or uneven sampling time points. The alignment was done such that the time corresponding to the highest values of the two observed curves overlap exactly. All statistical tests were two-tailed and were considered significant at p ≤ 0.05. Smooth trajectory analysis were performed using Prism 6 (GraphPad).

## Results

### HCV RNA and ALT activity during acute HCV infection

**Fig 1:**
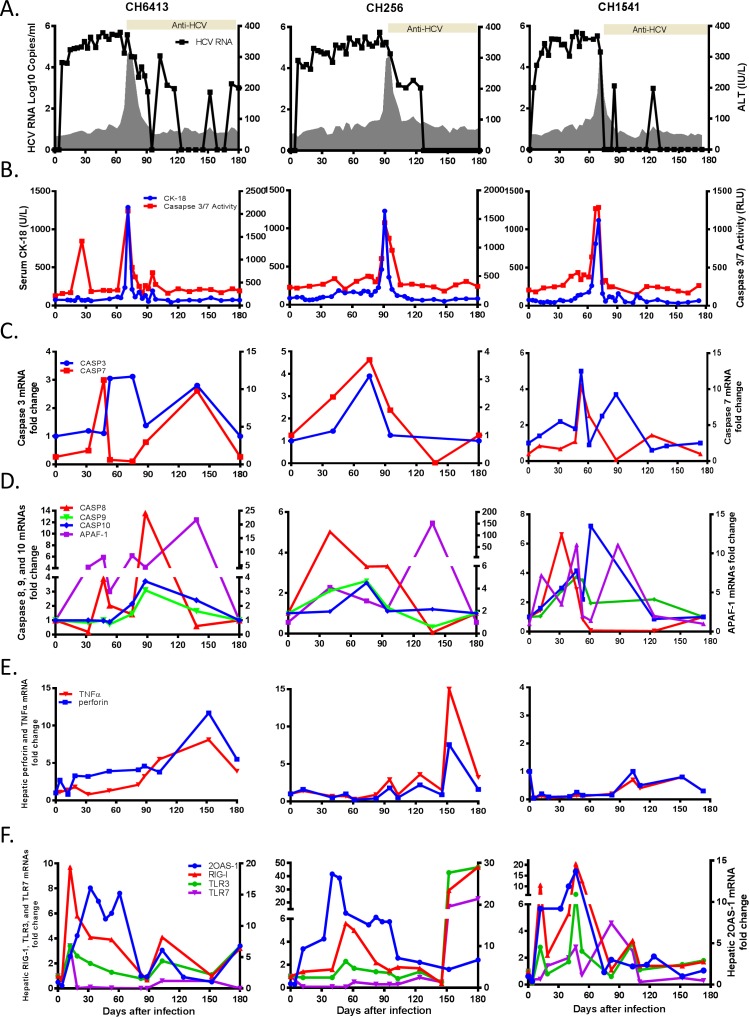
Apoptosis and expression of 2OAS-1, RIG-1, TLR3, and TLR7 mRNAs in livers of HCV-infected chimpanzees. (A), Course of infection in chimpanzees during acute HCV infection. Serum HCV RNA levels are represented as a line graph; serum ALT levels are indicated as grey shading; seropositivity for anti-HCV IgG is represented by the bar. (B), Serum caspase 3/7 activity (red line) and CK-18 levels (blue line) (C), Hepatic expression of caspase 3 (blue line) and 7 (red line) mRNAs (D), Levels of Caspase 8 (red line), Caspase 9 (green line), Caspase 10 (blue line), and APAF-1 (purple line) mRNAs in liver (E), Levels of TNFα (red line) and perforin (blue lines) mRNAs (F), Levels of 2OAS-1 (blue line), RIG-I (red line), TLR3 (green line), and TLR7 (purple line) mRNAs in liver. Each gene’s mRNA level was normalized to an endogenous reference gene and expressed as an increase over baseline liver tissue levels.

Three chimpanzees were infected with HCV genotype 1a and exhibited the typical pattern of HCV infection. HCV viremia was detected on day 4 in CH1541 and day 7 in CH256 and CH6413; the animals seroconverted between days 70 to 88 (Fig 1A). The peak level of HCV RNA was about 5.7 logs IU/ml. All three chimpanzees exhibited significant elevations of ALT activity that peaked between 70 and 95 days after inoculation and returned to baseline levels between days 88 to 153 after inoculation, at the time of serum HCV RNA decline. HCV RNA detection in CH6413 and CH256 continued until the end of the 180 day observation period, whereas CH1541 cleared HCV RNA by day 131.

### HCV infection induces apoptosis in chimpanzees with acute HCV infection

Caspase activation is a common feature of cell death through apoptosis. Caspase 3/7 activity levels in serum of all three chimpanzees were elevated from 71 to 91 days after inoculation and their peaks coincided with peak CK-18 levels (Fig 1B). Serum caspase 3/7 activity and CK-18 levels were closely correlated to ALT activity. Pearson correlation test confirmed that there is statistically significant strong positive correlation between caspase 3/7 activity, CK-18, and ALT activity (Table 1). In addition, in CH6413 and CH256, caspase 3 and 7 activities above-baseline cutoff levels were detected from day 110 to 180. Increased levels of caspase-3 and caspase-7 mRNAs in the liver were detected in all three animals, in which elevated mRNA expression was detected, from day 32 in CH1541, day 40 in CH256, and day 47 in CH6413. These levels peaked approximately 20 days before the highest levels of elevated caspase 3/7 activity in the corresponding serum samples (Fig 1B and 1C).

**Table 1:** Pearson correlation coefficients for association between HCV RNA, ALT activity and other markers in chimpanzees with acute HCV infection.

	HCV RNA		ALT activity	
Pearson correlation test	*r*	p value	*r*	p value
HCV RNA concentration	**1**		**0.219**	**0.035**
ALT activity	**0.219**	**0.035**	**1**	
Gene expression in liver				
RIG-I mRNA	**0.475**	**0.012**	-0.051	0.772
2OAS1 mRNA	**0.709**	**< 0.01**	0.207	0.184
TLR3 mRNA	0.198	0.322	-0.049	0.781
TLR7 mRNA	-0.242	0.223	-0.059	0.735
PD-L1 mRNA	0.284	0.088	0.127	0.392
CXCL10 mRNA	**0.496**	**0.005**	-0.099	0.556
CXCR3 mRNA	-0.328	0.077	-0.075	0.655
CCR1 mRNA	**-0.376**	**0.04**	-0.132	0.428
CCR7 mRNA	-0.077	0.687	-0.027	0.872
Caspase 3 mRNA	0.192	0.475	-0.068	0.795
Caspase 7 mRNA	0.072	0.791	-0.212	0.399
Caspase 8 mRNA	0.189	0.483	0.04	0.875
Caspase 9 mRNA	0.258	0.334	-0.061	0.809
Caspase 10 mRNA	0.168	0.534	0.021	0.935
APAF-1 mRNA	**0.515**	**< 0.01**	0.107	0.153
TNFα mRNA	-0.117	0.56	0.05	0.773
Perforin mRNA	-0.117	0.56	0.028	0.872
KIR2DL2/3 mRNA	**-0.55**	**0.003**	-0.17	0.328
NKG2D mRNA	-0.156	0.437	-0.035	0.84
PD-1 mRNA	**0.599**	**< 0.01**	0.018	0.917
CTLA4 mRNA	0.03	0.89	0.183	0.37
TIM3 mRNA	-0.034	0.878	0.153	0.445
IFNγ mRNA	-0.135	0.439	0.033	0.833
CD8β mRNA	-0.218	0.202	0.04	0.791
CD86 mRNA	-0.121	0.481	0.188	0.216
biomarkers in serum				
CK-18	0.201	0.098	**0.762**	**< 0.01**
Caspase 3/7 activity	0.26	0.071	**0.774**	**< 0.01**
soluble PD1	0.038	0.82	**0.645**	**< 0.01**
CXCL10	**0.71**	**0.001**	0.23	0.329
CXCL11	0.399	0.101	0.271	0.248
CCL5	0.324	0.189	0.153	0.518
CCL4	0.011	0.965	-0.047	0.845
CXCL9	0.002	0.995	0.209	0.377

All statistical tests were two-tailed and were considered significant at p ≤ 0.05.

High levels of initiator caspase-8, -9, -10, and adaptor molecule, APAF-1 mRNAs were also analyzed in the liver. In CH6413, expression of caspase 8, 9, 10, and APAF-1 mRNA was elevated from days 47, 88, 75, and 32, respectively (Fig 1D). In CH256, high levels of mRNA expression of caspase 8, 9, and APAF-1 were detected from days 40 to 75 and elevation of caspase 10 mRNA expression was detected at day 75. In CH1541, expression of caspase 8, 9, and 10 mRNAs was elevated from day 32 and APAF-1 mRNA expression was highly elevated from day 12 (7.2-fold change). Hepatic TNFα mRNA expression was elevated from day 82 to 180 in CH6413, on days 95, 124, and from day 152 to 180 in CH256 (Fig 1E). Hepatic perforin mRNA expression was elevated on day 4 and from day 12 to 180 and its peak level was detected on day 125 in CH6413. In CH256, perforin mRNA expression was elevated on days 124 and 152. However, elevated expression of TNFα and perforin mRNAs was not detected in CH1541.

### Expression of type I interferon and pattern recognition receptor gene (PRR) expression in the liver

Intrahepatic expression of 2′ 5′ oligoadenylate synthetase-1 (2OAS-1), type I interferon gene, and RIG-I, TLR3, and TLR7 as PRRs was analyzed. Elevation of 2OAS-1 mRNA expression was observed by day 12, coinciding with the appearance of serum HCV RNA, and peaked before serum ALT began to rise in all chimpanzees (Fig 1F). Increased levels of 2OAS-1 mRNA expression were maintained in CH6413 and CH256, but its expression had declined to baseline levels towards the end of acute-phase infection in CH1541.

In CH6413, hepatic expression of RIG-I and TLR3 mRNAs peaked on day 12 and remained elevated until day 32 or 53, and up-regulated again from day 103 to 180; TLR7 mRNA expression was elevated only on day 12 (Fig 1F). In CH256, hepatic expression of RIG-I mRNA and TLR3 mRNAs was elevated from days 53 to 82 and 53 to 61, respectively, and high levels of TLR7 mRNA expression were observed from days 152 to 180. In CH1541, RIG-I mRNA expression was elevated from days 12 to 53, and on day 103; expression of TLR3 mRNA was elevated on day 12, 47 to 53, and 103. High levels of TLR7 mRNA expression were detected on day 46, and from day 82 to 103.

### Hepatic expression of chemokines and natural killer cell activation markers

**Fig 2:**
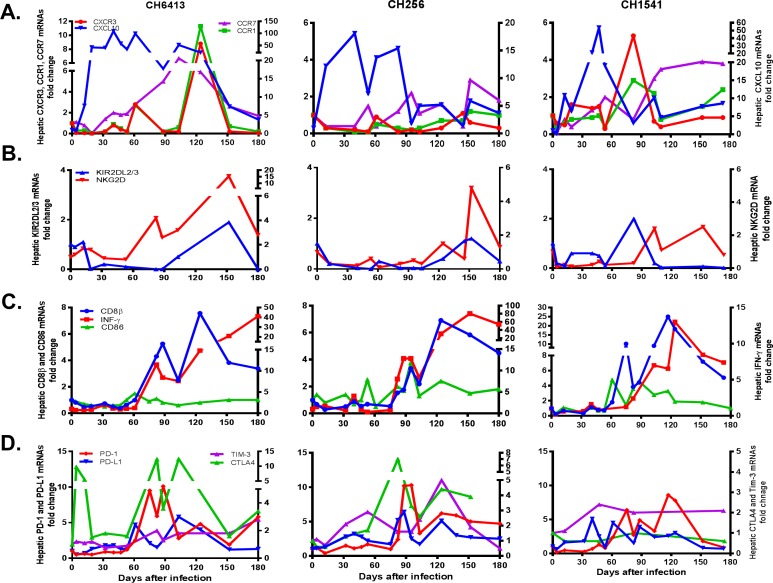
Expression of chemokines, NK cell and T cell co-activation and inhibition markers in liver. (A), Expression levels of chemokine mRNAs in the liver during acute HCV infection: CCR7 (purple line), CCR1 (green line), CXCR3 (red line), and CXCL10 (blue line). (B), Expression levels of NK cell marker mRNAs in the liver: KIR2DL2/3 (blue line) and NKG2D (red line). (C), T cell activation marker RNAs: CD8β (blue line), IFN-γ (red line), and CD86 (green line). (D), T cell inhibition marker mRNA expression in liver: PD-1 (red line), PD-L1 (blue line), Tim-3 (purple line), and CTLA4 (green line). Each gene’s mRNA level was normalized to an endogenous reference gene and expressed as an increase over baseline liver tissue levels.

Expression of chemokines, CXCR3, CCR1, CCR7, CXCL10, and NK cell activation marker, NKG2D, and NK cell inhibition marker, KIR2DL2/3 receptor mRNAs was analyzed to determine whether these genes were involved in induction of liver injury. Hepatic expression of CXCR3 and CCR1 mRNAs was elevated from day 61 to 124 in CH6413 and days 61 and 124 in CH1541, but not in CH256 (Fig 2A). High expression of CCR7 mRNA was detected from days 61 to 152 in CH6413, on days 95 and 152 in CH256, and days 61 to 152 in CH1541. Hepatic expression of CXCL10 mRNA was elevated from day 7 or 12 until the end of observation in CH6413 and CH1541, respectively, and on days 19 to 82, 103 to 124, and 152 to 180 in CH256. NKG2D mRNA was elevated between days 82 to 180 in CH6413, on day 152 in CH256, and between days 103 and 152 in CH1541, but KIR2DL2/3 receptor mRNA expression was not elevated in any of these chimps (Fig 2B).

### Expression of T cell activation and inhibition markers

T activation and inhibition markers were analyzed to determine their relationship to the liver injury. CD8β mRNA expression began to rise on days 82, 95 and 75 in CH6413, CH256, and CH1541, respectively, and peaked between days 118 and 124. High levels of CD8β mRNA expression were maintained until the end of acute phase of HCV infection in all chimpanzees. (Fig 2C). The level of IFNγ mRNA expression increased steadily as HCV viremia decreased and its levels were elevated from day 82 for CH6413 and CH256 until the end of observation. For CH256, IFNγ elevation was less marked than for the other two chimpanzees and levels declined toward the end of the observation period. High levels of hepatic PD-1 and PD-L1 mRNA expressions were found from days 61 to 180 and 40 to 180, respectively (Fig 2C). Up-regulation of PD-1 mRNA expression continued until the end of the observation period in CH6413 and CH256, while in CH1541, it had declined to baseline levels by day 124. The pattern of hepatic PD-L1 mRNA expression varied in all three chimpanzees. It fluctuated in CH1541 and CH6413 declining at the end of the acute infection. In CH256 it was still highly expressed at the end of acute infection. In all three chimpanzees, hepatic PD-L1 mRNA expression preceded rise in hepatic PD-1 mRNA expression. No significant changes in PD-1 and PD-L1 mRNA expression in PBMCs were observed in any chimpanzee, although slight increases were noted only occasionally in CH1541 (data not shown). CTLA4 mRNA expression was elevated from days 82 to 152 in CH6413 and CH256, but not in CH1541. High levels of Tim-3 mRNA expression were detected from days 47 to 180 in all three chimpanzees (Fig 2B). CD86 mRNA was elevated between days 53 and 124 in CH256 and CH1541, but that was not observed for CH6413 (Fig 2C).

### Soluble PD-1 and CXCL10 levels in serum: possible serum markers for detecting hepatic injury induced by acute HCV infection

**Fig 3:**
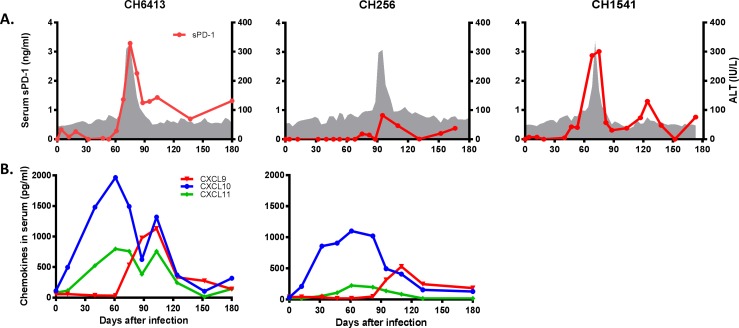
Serum levels of sPD-1 and chemokines. (A) Levels of serum sPD-1 (red line) were compared to ALT activity (gray shading). (B), serum levels of CXCL9 (red line), CXCL10 (blue line), and CXCL11 (green line) in CH6413 and CH256. Serum samples of CH1541 were not available so serum levels of chemokines by ELISA analysis could not be determined.

Baseline levels of sPD-1 were analyzed using serum samples from 10 naïve chimpanzees and 8 samples from the 3 study chimpanzees obtained before experimental inoculation. Levels of sPD-1 were measured at less than 0.2 ng/ml in all samples (data not shown). sPD-1 levels in CH6413 and CH1541 became elevated by days 47 and 61, respectively, peaking rapidly thereafter (Fig 3A). For CH256, the rise in sPD-1 levels was less marked. In CH6413 and CH256, levels of sPD-1 decreased after its peak, but sPD-1 levels were elevated until day 138 in CH1541. The level of sPD-1 in plasma correlated significantly with intrahepatic PD-1 mRNA (r = 0.491, p = 0.001) and serum ALT activity (r = 0.302, p = 0.035). Because of the lack of availability of serum samples from CH1541, levels of CXCL10 in serum were only determined in CH6413 and CH256. Baseline levels of CXCL10 were analyzed using 9 different pre-inoculation samples and determined to be 112 pg/ml in CH6413 and 32 pg/ml in CH256. High levels of circulating CXCL10 were detected from day 12 with 4.4-fold increase over baseline expression level in CH6413 and a 6.5-fold increase in CH256, and these levels peaked at day 61 in CH6413 and 82 in CH256 (Fig 3B).

### Correlation between hepatic mRNAs, HCV RNA concentration, and ALT activity levels

The Pearson correlation test was performed to determine the relationship between levels of hepatic mRNA expression, serum protein, and clinical parameters such as ALT activity and presence of HCV RNA during acute HCV infection. A weak correlation was detected between HCV RNA concentration and ALT activity. Serum HCV RNA levels were positively correlated with the expression of 2OAS-1, PD-1, APAF-1, RIG-I, PD-L1 and CXCL10 mRNAs and inversely correlated with CCR1 and KIR2DL2/3 mRNA expression (Table 1). Strong positive correlations were detected between ALT activity and CK-18, Caspase 3/7 activity, and soluble PD-1. These results indicate that hepatic expression of 2OAS-1, PD-1, APAF-1, RIG-I, PD-L1 and CXCL10 mRNAs was activated during HCV RNA replication. Statistically significant correlations were not detected between any of hepatic mRNAs and ALT activity.

### Lag correlation

**Fig 4:**
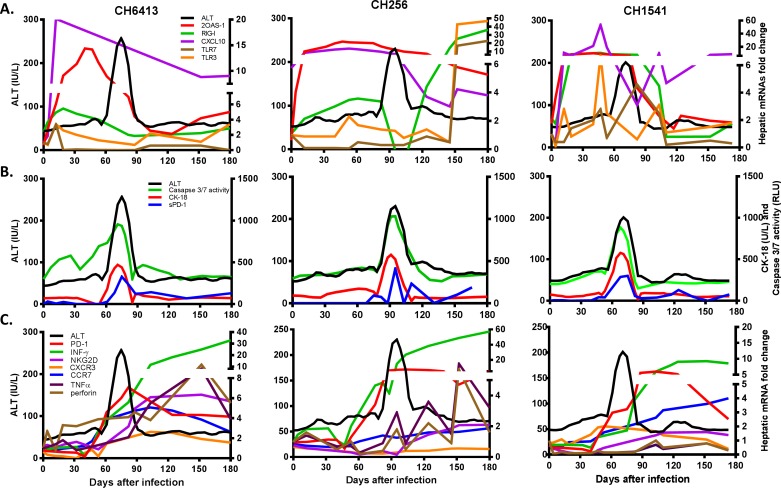
Smoothed trajectories of each hepatic mRNA expression compared to ALT activity during acute HCV infection. Hepatic expression of mRNAs was up-regulated before the peak (A), at the peak (B), and after the peak of ALT activity.

A simple linear correlation test such as the Pearson correlation only allows determination of linear relationships between two variables. Smooth trajectories as illustrated in Fig 4 provide a picture of trends in expression levels of different mRNAs and ALT during acute phase HCV infection. Trajectory curves were generated based on levels of hepatic mRNA expression that were up-regulated before the peak of ALT activity, at the peak, and after ALT activity peak in each chimpanzee (Fig 4). High levels of hepatic expression (≥ 2-fold change) of 2OAS-1, RIG-I, CXCL10, TLR3, and TLR7 mRNAs were detected before the ALT activity peak. Serum levels of CK-18, Caspase 3/7 activity, and soluble PD-1 were elevated at the peak of ALT activity, and hepatic expression of PD-1, IFNγ, NKG2D, perforin, CXCL3, TNFα, and CCR7 mRNAs was up-regulated after peak of ALT activity. Based on these trends, lag correlation testing was performed to determine the temporal relationship between these mRNAs and ALT activity (Table 2). ALT activity was positively correlated with RIG-I, TLR3, CXCL10, PD-L1, 2OAS-1, Caspase 7, and Caspase 10 mRNA expression from 3 to 12 days before the peak of ALT activity, indicating that the increase of these mRNA levels is associated with the subsequent increase in ALT activity. ALT activity was positively correlated with expression of markers for T cells and NK cells such as IFNγ, CD8β, CD86, KIR2D, and NKG2D mRNAs from 7 to 32 days after the peak of ALT activity, indicating that the expression of these genes was up-regulated after elevation of ALT activity.

**Table 2:** Lag correlation coefficients between ALT activity and other markers based on the Pearson correlation model.

mRNA markers	Lag time in days	*r*	*p*
**RIG-1 mRNA**	**14**	**0.1597**	**0.0391**
TLR3 mRNA	0	-0.1959	0.0082
TLR7 mRNA	0	-0.2126	0.0041
**CXCL10 mRNA**	**10**	**0.533**	**< 0.001**
**PD-L1 mRNA**	**10**	**0.5324**	**< 0.001**
**2OAS-1 mRNA**	**31**	**0.7713**	**< 0.001**
CASP3 mRNA	-53	0.4865	< 0.001
**CASP7 mRNA**	**24**	**0.2634**	**0.0009**
CASP8 mRNA	-17	0.6791	< 0.001
CASP9 mRNA	-17	0.4554	< 0.001
**CASP10 mRNA**	**10**	**0.9063**	**< 0.001**
CXCL3 mRNA	-53	0.6199	< 0.001
CCR1 mRNA	-53	0.7496	< 0.001
CCR7 mRNA	-17	0.4577	< 0.001
IFNγ mRNA	-109	0.4555	0.0001
CD86 mRNA	-24	0.634	< 0.001
CD8β mRNA	-46	0.8775	< 0.001
PD-1 mRNA	-24	0.713	< 0.001
CTLA4 mRNA	-32	0.6516	< 0.001
Tim-3 mRNA	-53	0.8159	< 0.001
KIR2DL2/3 mRNA	-74	0.6412	< 0.001
NKG2D mRNA	-81	0.8001	< 0.001
perforin mRNA	-81	0.6256	< 0.001
TNFα mRNA	-81	0.6856	< 0.001

## Discussion

Experimentally infected chimpanzees allowed us to analyze the kinetics of host response induced by acute HCV infection. Profiles of innate and adaptive immunologic responses and those relating to cell death signaling biomarkers expressed in liver and serum provided insights into early immune responses associated with liver injury during acute HCV infection. We found that apoptosis markers, caspase 3/7 activity and CK-18, were positively correlated with ALT activity and elevation of hepatic caspase 3 and 7 mRNA expression was detected earlier than serum caspase 3/7 activity and M30 antigen. These results demonstrated evidence for association between apoptosis and liver damage during acute HCV infection. Two apoptotic pathways, intrinsic and extrinsic, are converging on activation of effector caspases but require different initiation caspases to start the process. The intrinsic apoptosis pathway occurs through cytochrome c released after mitochondrial damage, activation of initiator caspases, caspase 9 and APAF-1, and subsequent effector caspase 3 and caspase 7 activation [32]. For extrinsic apoptosis, TNFα, a pleiotropic pro-inflammatory cytokine produced mainly by activated macrophages, can initiate apoptosis through death receptors in hepatocytes leading to subsequent liver injury [33]. We found that caspase 9 and adaptor molecule, APAF-1 mRNA expression was detected in all three chimpanzees and APAF-1 mRNA expression was elevated as early as 12 days after inoculation (Fig 1D) and elevation of TNFα mRNA expression was detected either after the peak of ALT activity (days 82 or 95 in CH6413 and CH256) or not detected (CH1541). These results suggest that apoptosis could occur through the mitochondrial-mediated, intrinsic pathway rather than the extrinsic pathway during the acute phase of HCV infection. A previous study reported that HCV infection induced mitochondrial-mediated apoptosis pathway in which the increased accumulation of the proapoptotic protein Bax, resulted in release of cytochrome c after mitochondrial swelling and activation of caspase 3 in HCV-infected Huh 7.5 cells [34].

Innate immune responses provide the first line of defense against HCV infection. Type I IFN, IFNα/β, and type III IFN, IFN-λ, responses induced similar sets of interferon-stimulated genes (ISG) [35]. Up-regulation of type III IFN, IL-28 and IL-29 mRNA were detected in liver and increased levels of IL-28 and IL-29 protein in serum of chimpanzees with acute HCV infection [20, 21]. RIG-I, TLR3, and TLR7, and induce interferons, cytokines, and chemokines during HCV infection [36, 37]. RIG-I mediated IFN response was shown to play an important role in controlling virus infection in HCV-infected cells [38]. In other reports, RIG-I signaling and TLR-3 signaling are required for CXCL10 induction in hepatocytes during early HCV infection but type I or type III IFNs are not required in the initial induction phase [39]. These *in vitro* infection studies were conducted in an independent system but inflammatory and antiviral pathways operate simultaneously to control virus infection [40]. We also observed that up-regulation of hepatic expression of 2OAS-1, RIG-I, TLR3, TLR7, and CXCL10 mRNAs was found as early as 7 days after infection and coincident with serum HCV RNA appearance (Figs 1 and 2). These results indicated that PRRs-mediated antiviral pathways, type I and type III IFNs responses, and proinflammatory chemokines are produced by HCV-infected hepatocytes at the same time and establish a strong antiviral state in chimpanzees during the acute hepatitis C infection.

IFN-inducible gene, 2OAS-1, proinflammatory cytokines, and chemokines are involved in eliminating viral infection from infected cells and induce apoptosis of infected cells. HCV-mediated activation of RNase L, 2OAS-dependent ribonuclease, leads to apoptosis and elimination of HCV-infected cells. Apoptosis initiated by RNase L is characterized by the release of cytochrome c from mitochondria and requires caspase 3 activity [41]. TLR3 can directly trigger apoptosis in human cancer cells [42]. CXCL10 expression was correlated with liver cell apoptosis in humans and mice, and apoptosis occurs through noncognate receptor TLR4 [36]. Up-regulation of CXCL10 expression was observed in patients who inject drugs and associated with subsequent increase in ALT levels during acute hepatitis C infection [37]. In our study, hepatic 2OAS-1 mRNA was positively correlated with caspase 3/7 activity (r = 0.504, p = 0.02), hepatic RIG-I mRNA expression was positively correlated with caspase 3 mRNA expression (r = 0.669, p = 0.034), and CXCL10 mRNA expression was positively correlated with caspase 7 mRNA expression (r = 0.711, p = 0.014). We also observed elevation of ALT activity after increases in each of these mRNA levels. Expression profiles of these genes indicated that early expression of 2OAS-1, RIG-I, TLR3, and CXCL10 was associated with increases in HCV RNA levels, and followed elevation of ALT activity at 10 to 12 days before the peak of ALT activity (Table 2). These results suggest that 2OAS-1, RIG-I, TLR3, and CXCL10 are involved in inducing apoptosis upon HCV infection and lead to liver injury in acute HCV infection.

NK cells and CD8+ T cells were shown to be involved in elimination of HCV infected hepatocytes [43, 44]. In our study, expression of NKG2D and KIR2D mRNAs, hepatic NK cell markers, markers for T cell activation (CD86, CD8β, and IFNγ) and T cell inhibition markers (PD-1, CTLA4) were up-regulated after the peak of ALT activity in all three chimpanzees (Fig 2). Lag correlation analysis showed NK cell markers, T cell activation/inhibition markers were negatively correlated with ALT activity. T cell and NK cell-induced hepatocyte apoptosis was mediated largely by members of the TNF receptor family including TNFα, Fas, and TRAIL [45]. We found that elevation of TNFα mRNA expression was detected either after the peak of ALT activity or not elevated, suggesting that elimination of HCV infected hepatocytes by T cells and NK cells may not lead to elevation of ALT activity in experimentally infected chimpanzees with acute HCV infection.

Increase in serum levels of caspase 3/7 activity, CK-18, and soluble PD-1 have strong positive correlation with ALT activity during acute HCV infection (Fig 4). Currently serum ALT activity is used as marker for liver injury but it cannot distinguish between acute and chronic HCV infection. Acute hepatitis C is defined by the presence of any sign or symptom of acute viral hepatitis plus either jaundice or elevated ALT activity, with presence of anti-HCV antibody or HCV RNA [3]. However, most people with acute HCV infection are asymptomatic at time of diagnostic testing and already positive for anti-HCV antibody. The chimpanzee model of HCV infection allows host responses to be analyzed closely, particularly during the early stage of infection. Previously we reported that serum levels of miR-122 were positively correlated with ALT activity during acute HCV infection [6]. Serum CK-18 levels showed high sensitivity and specificity in diagnosing non-alcoholic steatohepatitis in patients with non-alcoholic fatty liver disease and reflected clinical disease more accurately than ALT levels [46, 47]. A meta-analysis of elevated serum cytokeratin-18 levels in hepatitis showed its clinical value for identifying the development of hepatitis [48]. The biomarkers such as CK-18, caspase 3/7 activity, sPD-1, and serum miR-122 levels could be used as adjunctive criteria to diagnose acute hepatitis C more definitively and to provide a sharper case definition for acute hepatitis C.

In summary, we found that cell death signaling is initiated by early induction of RIG-I, TLR3, and CXCL10 mRNAs and type I IFN response causing elevation of ALT activity, and that initiation of this signaling pathway occurs before the activation of NK and T cells during acute HCV infection.

## Supporting Information

S1 Materials and Methods (DOCX)
