# Supplementary material for: Elevation of Alanine Aminotransferase Activity Occurs after Activation of the Cell-Death Signaling Initiated by Pattern-Recognition Receptors ‎but before Activation of Cytolytic Effectors in NK or CD8+ T Cells in the Liver During Acute HCV Infection
Source: PLoS One. 2016 Oct 27;11(10):e0165533. doi: 10.1371/journal.pone.0165533 (PMC5082795; doi:10.1371/journal.pone.0165533)
Supplement: S1 Materials and Methods — (DOCX) [file pone.0165533.s001.docx]

**Supporting Information**

**S1. Supporting materials and methods**

Primers and Probes used for CASP3 (XM009448539), CASP7 (XM508043), CASP8 (XM009443995), CASP9 (XM513049), CASP10 (XM006712796), and APAF-1 (XM001151719) real-time PCR were custom designed based on GenBank sequence:

The PCR primer sequences for caspase-3 gene were: forward, 5′-TT TTT CAG AGG GGA TCG TTG-3′; reverse, 5′-CGGCC TCCACTGGTATTTTA-3′; caspase-7 gene: forward, 5′- AGTGA CAGGTATGGGCGTTC-3′; reverse, 5′-CGGCATTTGTATGGTCCTCT-3′. caspase-8 gene: forward, 5′- AAGCAAACCTCGGGGATACT-3′; reverse, 5′- GGGGCTTGATCTCAAAATGA -3′, caspase-9 gene: forward, 5′- CTAGTTTGCCCACACCCAGT -3′; reverse, 5′- GCATTAGCGACCCTAAGCAG -3′, caspase-10 gene: forward, 5′- GACGCCTTGATGCTTTCTTC -3′; reverse, 5′- ATGAAGGCGTTAACCACAGG -3′,APAF-1, forward, 5′- TTCTGATGCTTCGCAAACAC-3′; reverse, 5′-CTGGCAAATCTGCCTTCTTC-3′.

Primers and probes for, 2′ 5′ oligoadenylate synthetase-1 (2,5-OAS-1) (Hs00242943_m1), RIG-1 (Hs01061436_m1), TLR3 (Hs01551078_m1), TLR7 (Hs01933259_s1), TNFα (Hs00174128_m1), CXCL10 (Hs01124252_g1), perforin (Hs00169473_m1), CXCR3 (Hs01847760_s1), CCR1 (Hs00928897_s1), CCR7 (Hs01013469_m1), NKG2D (Hs00183683_m1), KIR2D (Hs03407415_gH), CD8β (Hs00174762_m1), CD86 (Hs01567025_m1), and IFNγ (Hs00174143_m1), PD-1 (Hs00169472_m1), PD-L1 (Hs00204257_m1), GAPDH (4325317E), CTLA-4 (Hs01011591_ml), Tim-3 (Hs00262170_m1) were purchased from Life Technologies (Grand Island, NY).

**Soluble PD-1 in serum/plasma:**

Microtiter plates were pre-coated with antibody against PD-1 (2 mg/L) (R&D systems Inc., Minneapolis, MN) and incubated overnight at 4°C. The plates were then washed 4 times with wash buffer (10 mM phosphate buffer, pH 7.4, 150 mM NaCl and 0.05% Tween 20). Blocking buffer (10 mM phosphate buffer, pH 7.4, and 1% FBS) was then added. Serum or plasma (50 μl/well) was added to wells in triplicate. A PD-1 fusion protein (0.78 to 100 ng/ml) (PD-1-Fc; R&D Systems Inc.) was used as a standard. The plates were incubated for 2 hr at 37°C, and after washings for 3 times, biotinylated anti-human PD-1 (R&D Systems Inc.) (35 ng/ml) was added and incubated for an additional 2 hr at 37°C. After washing, streptavidin-HRP conjugate was added according to manufacturer’s recommendation (R&D Systems Inc.). Color reactions were developed using 3′3′, 5′5′-tetramethylbenzidine substrate and subsequently stopped with 2N sulfuric acid according to manufacturer’s recommendation (R&D Systems Inc). Absorbance was measured at a wavelength of 450 nm.
